# Reducing psychotropic medication use in patients with dementia: the impact of multimodal care‐communication skill training for medical staff in long term care hospital

**DOI:** 10.1002/alz.088939

**Published:** 2025-01-09

**Authors:** Miwako Honda, Masaki Kobayashi

**Affiliations:** ^1^ National Hospital Organization Tokyo Medical Center, Tokyo Japan; ^2^ Unitiy Hospital, Rochester, NY USA

## Abstract

**Background:**

Psychotropic drug prescriptions are commonly used to manage behavioral and psychological symptoms of dementia in elderly patients in long‐term care facilities. The prevalence of psychotropic drug use in this population raises concerns due to potential side effects, polypharmacy and quality of life of the patients.

**Aim:**

To assess the trends in psychotropic drug prescriptions for elderly patients with dementia following the continuous implementation of multimodal comprehensive care communication skills training for staff in a long‐term care hospital.

**Method:**

This retrospective single‐centre cross‐sectional study utilized the database of an urban public long‐term care hospital, from 2016 to 2020. The study involved 130 hospital staff, comprising 52 nurses, 48 professional caregivers, 7 rehabilitation staff, 3 physicians, and 3 pharmacists. All staff members underwent a French origin multimodal comprehensive care communication skill training (Humanitude) from October 2014 to December 2015, followed by continuous monthly training sessions until the conclusion of the study in 2020. Antipsychotics prescription rates for patients aged over 65 years with dementia were measured throughout the study period. No significant changes in the proportion nor severity of patients' disease backgrounds were observed during the study period.

**Results:**

A total of 506 eligible patients were identified, with a mean age of 86.0 years (IQR 81.0‐90.0), and 283 (55.9%) were women. The prescription rates for psychotropic drugs among residents with dementia showed a significant decrease (43.5% in 2016, 27.0% in 2020, p = 0.01). Notably, the prescription rates for anxiolytics demonstrated a significant reduction (from 4.7% to 0.0%), while rates for antipsychotic drugs, hypnotics, antidepressants, antiepileptic drugs, and lithium remained unchanged over time. The prescription rates for antidementia drugs significantly decreased from 15.3% to 4.0%.

**Conclusion:**

The study found a significant reduction in the prescription rates of psychotropic drugs after implementing multimodal comprehensive care communication skills training for staff at a long‐term care hospital. This outcome indicates that providing skill training to hospital staff is associated with a decrease in behavioral and psychological symptoms of dementia among patients, resulting in a reduced need for psychotropic drugs. The enhanced communication skills among long‐term care staff demonstrate a tangible impact on reducing drug use among patients with dementia.

